# Vagus Nerve Stimulation Therapy for the Treatment of Seizures in Refractory Postencephalitic Epilepsy: A Retrospective Study

**DOI:** 10.3389/fnins.2021.685685

**Published:** 2021-08-19

**Authors:** Yulin Sun, Jian Chen, Tie Fang, Lin Wan, Xiuyu Shi, Jing Wang, Zhichao Li, Jiaxin Wang, Zhiqiang Cui, Xin Xu, Zhipei Ling, Liping Zou, Guang Yang

**Affiliations:** ^1^Department of Pediatrics, Chinese PLA General Hospital, Beijing, China; ^2^Department of Pediatrics, The First Medical Center, Chinese PLA General Hospital, Beijing, China; ^3^Department of Functional Neurosurgery, Beijing Children’s Hospital, Capital Medical University, National Center for Children’s Health, Beijing, China; ^4^The Second School of Clinical Medicine, Southern Medical University, Guangzhou, China; ^5^Department of Neurosurgery, Chinese PLA General Hospital, Beijing, China

**Keywords:** vagus nerve stimulation, postencephalitic epilepsy, refractory epilepsy, encephalitis, children

## Abstract

**Background:**

Vagus nerve stimulation (VNS) has been demonstrated to be safe and effective for patients with refractory epilepsy, but there are few reports on the use of VNS for postencephalitic epilepsy (PEE). This retrospective study aimed to evaluate the efficacy of VNS for refractory PEE.

**Methods:**

We retrospectively studied 20 patients with refractory PEE who underwent VNS between August 2017 and October 2019 in Chinese PLA General Hospital and Beijing Children’s Hospital. VNS efficacy was evaluated based on seizure reduction, effective rate (percentage of cases with seizure reduction ≥ 50%), McHugh classification, modified Early Childhood Epilepsy Severity Scale (E-Chess) score, and Grand Total EEG (GTE) score. The follow-up time points were 3, 6, and 12 months after VNS. Pre- and postoperative data were compared and analyzed.

**Results:**

The median [interquartile range (IQR)] seizure reduction rates at 3, 6, and 12 months after VNS were 23.72% (0, 55%), 46.61% (0, 79.04%), and 67.99% (0, 93.78%), respectively. The effective rates were 30% at 3 months, 45% at 6 months, and 70% at 12 months. E-chess scores before the operation and at 3, 6, and 12 months after the operation were 10 (10, 10.75), 9 (9, 10), 9 (9, 9.75), and 9 (8.25, 9) (*P* < 0.05), respectively. GTE scores before surgery and at 12 months after the operation were 11 (9, 13) and 9 (7, 11) (*P* < 0.05), respectively. The mean intensity of VNS current was 1.76 ± 0.39 (range: 1.0–2.5) mA. No intraoperative complications or severe post-operative adverse effects were reported.

**Conclusions:**

Our study shows that VNS can reduce the frequency and severity of seizure in patients with refractory PEE. VNS has a good application prospect in patients with refractory PEE.

## Introduction

Encephalitis in early childhood may damage brain tissue and it may cause neurological sequelae, including epilepsy. Epidemiological studies reported a 16.4–29% incidence of epilepsy secondary to encephalitis, among which refractory epilepsy accounts for 7–15% (Lee et al., 2007; Sellner and Trinka, 2012; Singh et al., 2015; Pillai et al., 2016). Most cases of postencephalitic epilepsy (PEE) are intracatable and cannot be treated by traditional epilepsy surgery because of the diffuse epileptogenic foci, which are difficult to localize. The severity and refractory nature of PEE necessitate the identification of alternative treatment options. Since it was first proposed in 1988, vagus nerve stimulation (VNS) has been recognized worldwide as an approved and effective adjunctive therapy for medically intractable epilepsy (Hajnsek et al., 2005; González et al., 2019). However, there are few reports on the treatment of refractory PEE by VNS, especially in children. To better evaluate the efficacy of VNS for PEE, we conducted an observational study of 20 patients under 18 years old, summarizing their clinical characteristics and long-term prognosis.

## Materials and Methods

All patients who were implanted with a stimulator (G111 or G112, Pins Medical, Ltd., Beijing, China) with remote programming function between August 2017 and October 2019 were included in our retrospective study, and they completed at least 12 months of follow-up. Patients at the Chinese PLA General Hospital and Beijing Children’s Hospital were diagnosed according to the practical clinical definition of the International Anti-Epilepsy Alliance (ILAE) (Fisher et al., 2017), which defines PEE as the occurrence of at least one unprovoked seizure after the acute phase of encephalitis. All patients had been diagnosed as having drug-resistant epilepsy (DRE), which is defined by the ILAE as the persistence of seizures at final follow-up despite the use of at least two appropriate anti-seizure medications (ASMs) (Fisher et al., 2017; Kimizu et al., 2018). Classification of seizure type was performed by at least two pediatric neurologists in accordance with the 2017 ILAE classification proposal based on clinical symptoms and EEG findings. The VNS stimulator was implanted by neurosurgeons according to standard procedures (Révész et al., 2016). The stimulator was turned on within 2 weeks after implantation, and electrical stimulation was commenced at 0.2–0.5 mA depending on patient tolerance (signal frequency: 30 Hz, pulse width: 250 or 500 μs, duty cycle: 30 s on/3 or 5 min off). The stimulation parameters were modified every 4 weeks in the first 3 months in accordance with observed therapeutic effects and adverse reactions. Parameters were changed according to each patient’s situation. When the treatment effect was not good, we increased the output current and stimulation time and reduced the intermission time. When the adverse reaction was obvious, we decreased the output current and stimulation time and increased the intermission time. The current amplitude of each adjustment was between 0.1 and 0.5 mA. Additionally, each patient was given a handheld magnet to enhance stimulation before seizure onset or during the seizure. The stimulation amplitude associated with the magnet mode was 0.2 mA higher than that associated with the normal mode, with other stimulation parameters kept constant.

Follow-up was performed at 3, 6, and 12 months after implantation. We collected data including (1) demographics; (2) age at onset; (3) encephalitis etiology; (4) seizure type; (5) seizure frequency; (6) number and dosage of ASMs; (7) VNS parameters; (8) improvement of cognitive, linguistic, and athletic ability; (9) post-operative adverse effects; and (10) electroencephalogram (EEG) findings. Informed consent was obtained from the children or their guardians for the purpose of this study.

Vagus nerve stimulation efficacy was evaluated based on the seizure reduction, effective rate, McHugh classification, modified Early Childhood Epilepsy Severity Scale (E-Chess), and Grand Total EEG (GTE) score. Seizure reduction was assessed as follows: (baseline frequency − frequency with VNS)/(baseline frequency) × 100%. It was defined as NA (not available) if the value was negative, which meant the seizure frequency increases. The baseline frequency was recorded for 2 weeks before VNS operation (Riikonen, 2020). Effectiveness was defined as seizure reduction ≥ 50%, and the effective rate was defined as the percentage of responders with a seizure reduction rate was ≥50% (Colicchio et al., 2010). The McHugh classification was used to further evaluate the VNS efficacy (McHugh et al., 2007): Class IA (most obvious improvement) to Class V (no improvement). Seizure severity was assessed using a modified E-chess score (Humphrey et al., 2008; Ho et al., 2018), which was calculated based on the following four severity indices: time period over which seizures occur, number of seizure types, number of ASMs used, and response to treatment. The score ranged from 3 (least severe) to 12 (most severe). The McHugh classification and the modified E-chess score were independently evaluated by two pediatric neurologists. If scores were inconsistent, they discussed and determined final scores. EEG abnormalities were evaluated using the GTE score (Limotai et al., 2018), which was calculated based on six items: frequency of rhythmic background activity, diffuse slow-wave activity, reactivity of rhythmic background activity, paroxysmal activity, focal abnormalities, and sharp-wave activity. The score ranged from 1 (normal EEG) to 31 (most severe). The GTE scores were scored independently by two EEG experts and were discussed and determined if they were inconsistent. We also recorded improvement in cognitive, linguistic, and athletic abilities at 12 months after the operation, based on subjective feeling and descriptions given by the patients or their guardians. The development level was also evaluated, using the Gesell Developmental Schedules (GDS), but only in two patients (both aged < 6 years).

SPSS 22.0 statistical software (IBM Corp., Armonk, NY, United States) was used for data analysis. Categorical data are expressed as percentages, while the continuous data are expressed as mean ± standard deviation (SD) (range) if they were normally distributed and as median [interquartile range (IQR)] if non-normally distributed. The Shapiro–Wilk test was used to determine whether data were normally distributed. Non-parametric test (Wilcoxon signed-rank test or Friedman M test) was used to evaluate the differences of GTE score and E-chess score at each follow-up point and baseline. Fisher exact test, *t*-test or Mann–Whitney *U* test were used to compare the differences of onset age, operation time and other factors between the effective group and the ineffective group at 12 months after VNS. Bonferroni correction was applied for multiple comparison. *P* < 0.05 was considered significant.

## Results

Thirteen male and seven female participants who were diagnosed with refractory PEE were included in this retrospective study. The age of 20 patients ranged from 2 to 17 years. The age at encephalitis onset was 1.75 (0.68, 6) (range: 0.01–11) years. Thirteen (65%) patients had experienced viral encephalitis, two (10%) had experienced bacterial meningoencephalitis, and five (25%) had experienced unknown etiology encephalitis. The age at epilepsy onset after encephalitis was between 0.33 and 12 years, with a mean age of 4.22 ± 3.64 years. Ten (50%) patients had generalized seizures, eight (40%) had focal seizures, and two (10%) patient had both focal and generalized seizures. The time from encephalitis to epilepsy was 0.06 (0, 1.09) (range: 0–4.83) years. The mean age at VNS operation was 6.65 ± 4.83 (range: 1.42–15) years old. At the time of VNS operation, patients had experienced seizures for 1.42 (1, 2.92) (range: 0–10) years. The clinical characteristics of these 20 patients are given in Supplementary Table 1.

**FIGURE 1:**
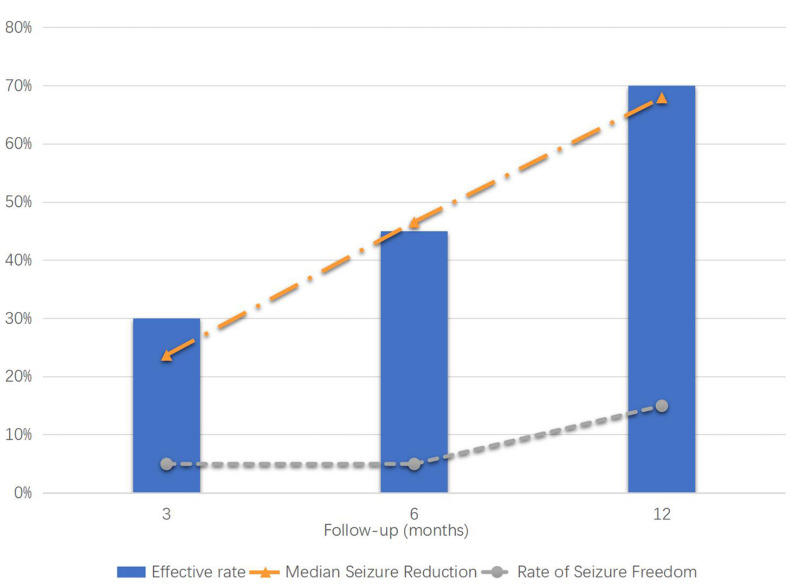
Median seizure reduction, effective rate, and rate of seizure freedom (%).

**FIGURE 2:**
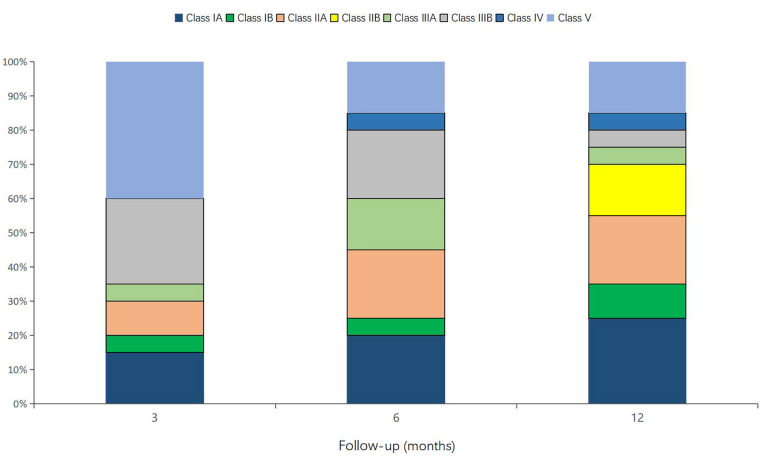
McHugh classification percentage (%).

**FIGURE 3:**
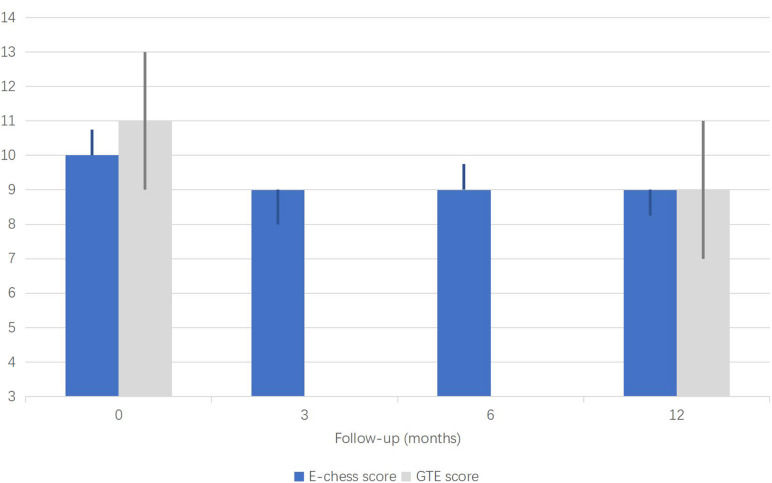
Median E-chess score and GTE score (median, interquartile range).

The types of seizure of patient 7 at 12 months after operation changed from tonic and emotional seizure to tonic seizure, and the number of antiepileptic drugs also changed from five to three. The classification of seizures and the number of antiepileptic drugs did not change in other patients during the follow-up period. The median (IQR) seizure reduction rates at 3, 6, and 12 months after VNS operation were 23.72% (0, 55%), 46.61% (0, 79.04%), and 67.99% (0, 93.78%), respectively. There was no statistical difference at any of the follow-up time points relative to baseline. The effective rate was 30% at 3 months, 45% at 6 months, and 75% at 12 months after the operation (Table 1 and Figure 1). Furthermore, 60, 80, and 80% of patients experienced Class III outcomes and above at 3, 6, and 12 months after the operation (Table 1 and Figure 2). The modified E-chess score before the operation was 10 (10, 10.75), and the scores at 3, 6, and 12 months after the operation were 9 (9, 10), 9 (9, 9.75), and 9 (8.25, 9) (*P* = 0.004, *P* < 0.001, and *P* < 0.001 compared with the baseline), respectively (Table 1 and Figure 3). There was significant difference between the follow-up time points and the baseline, but there was no significant difference among the follow-up time points. The GTE scores of 20 patients before the operation and at 12 months (seven patients who did not have an EEG examination at 12 months were excluded) were 11 (9, 13) and 9 (7, 11) (*P* = 0.022) (Table 1 and Figure 2).

**TABLE 1:** Efficacy of VNS for each patient.

No.	Parameters at 12 months after VNS	Seizure reduction (%)			McHugh classification			E-chess score				GTE	
	Current, pulse width, frequency, stimulation time, interval time	3 months	6 months	12 months	3 months	6 months	12 months	Before VNS	3 months	6 months	12 months	Before VNS	12 months
1	1.6 mA, 250 μs, 30 Hz, 30 s, 5 min	50	62.5	62.5	IIA	IIA	IIA	11	10	10	10	12	8
2	1.5 mA, 500 μs, 30 Hz, 30 s, 5 min	0	65.38	65.38	V	IIA	IIB	10	10	9	9	11	9
3	1.7 mA, 250 μs, 30 Hz, 30 s, 5 min	45.45	27.27	45.45	IIIB	IIIA	IIIA	10	9	8	8	13	12
4	1.7 mA, 500 μs, 30 Hz, 60 s, 1.8 min	0	0	0	V	V	V	11	11	11	11	10	9
5	1.3 mA, 250 μs, 30 Hz, 30 s, 5 min	30.77	73.08	100	IIIB	IIA	IA	10	9	9	8	13	6
6	1.0 mA, 500 μs, 30 Hz, 30 s, 5 min	95.56	95.56	95.56	IB	IB	IB	11	8	8	8	6	5
7	1.0 mA, 250 μs, 30 Hz, 30 s, 5 min	85	85	NA	IA	IA	V	10	9	9	9	9	11
8	2.0 mA, 500 μs, 30 Hz, 30 s, 3 min	16.67	16.67	50	IIIB	IIIA	IIA	10	9	9	9	10	10
9	2.0 mA, 500 μs, 30 Hz, 30 s, 3 min	33.33	55.56	77.78	IIIB	IIA	IIA	11	10	10	10	NA	NA
10	2.0 mA, 500 μs, 30 Hz, 30 s, 3 min	0	0	50	V	V	IIB	10	10	10	9	15	12
11	1.8 mA, 500 μs, 30 Hz, 30 s, 5 min	0	0	0	V	IV	IV	10	10	9	9	NA	NA
12	2.0 mA, 500 μs, 30 Hz, 30 s, 1.14 min	15.38	46.15	46.15	IIIB	IIIB	IIIB	10	9	9	9	6	4
13	2.0 mA, 500 μs, 30 Hz, 30 s, 1.8 min	0	0	0	V	V	V	10	10	10	10	9	NA
14	1.3 mA, 500 μs, 30 Hz, 30 s, 3 min	0	40	92	V	IIIB	IB	10	9	9	9	NA	NA
15	2.2 mA, 250 μs, 30 Hz, 30 s, 5 min	100	100	100	IA	IA	IA	10	8	8	8	NA	NA
16	2.0 mA, 500 μs, 30 Hz, 30 s, 3 min	47.06	47.06	70.59	IIIA	IIIA	IIA	10	9	9	9	13	NA
17	1.8 mA, 500 μs, 30 Hz, 30 s, 1.8 min	94.8	96.8	96.8	IA	IA	IA	11	9	9	9	18	11
18	1.8 mA, 500 μs, 30 Hz, 30 s, 5 min	60	90	100	IIA	IA	IA	10	8	8	7	NA	NA
19	2.0 mA, 500 μs, 30 Hz, 30 s, 1.8 min	0	40	72	V	IIIB	IIB	10	9	9	9	12	11
20	2.5 mA, 500 μs, 30 Hz, 30 s, 1.1 min	0	20.45	90.91	V	IIIB	IA	10	9	9	9	8	9

With regard to improvements of cognitive, linguistic, and athletic abilities at 12 months after the operation, we found that seven patients had improvements in all three domains; two patients had improved cognitive and athletic ability; one patient had improved cognitive and linguistic ability; three patients had only improved cognitive ability; one patient had only improved athletic ability; and the remaining six patients showed no obvious improvements. Patients 1 and 5 underwent GDS evaluation, and their developmental quotient for each ability increased by 2–16 points. The clinical efficacy of 20 patients is given in Supplementary Table 1. Supplementary Table 2 shows the distribution difference between the effective group and the ineffective group 12 months after VNS operation. There were no statistical differences in gender, the type of encephalitis, the type of seizure, the encephalitis onset age, the epilepsy onset age, the age at implantation, the time from encephalitis to epilepsy, and the time from epilepsy to VNS operation between effective group and ineffective group.

During follow-up, the VNS stimulation system for all 20 patients was found to work normally. By the last follow-up, each of the 20 patients had undergone VNS adjustments 3–10 times, and the interval between each adjustment was 4–12 weeks. At the last follow-up, the mean current intensity at the time of last follow-up was 1.76 ± 0.39 (range: 1.0–2.5) mA. The pulse width was 250 or 500 μs, the frequency was 30 Hz, and the stimulation time and interval time were adjusted for six patients. Specific adjustment parameters are shown in Table 1.

None of the patients had any serious adverse reactions. Only patient 4 and patient 6 experienced coughing (as a transient response to electrical stimulation), which was tolerable. No patients required to stop stimulation due to adverse reactions.

## Discussion

Postencephalitic epilepsy is a common sequela of acute encephalitis, which is defined as unprovoked seizure occurring after the acute phase of encephalitis. In four out of 20 patients, the epilepsy diagnosis occurred more than a year, and up to 4.83 years, after the diagnosis of encephalitis. Actually, when they were diagnosed with epilepsy, we carried out comprehensive examinations of the patients, excluding the causes of infection, immunity, metabolism, and heredity. Combined with the history, we considered that it was related to the structural damage caused by previous encephalitis. Research has shown that nearly half of all children with PEE will eventually develop DRE (Lee et al., 2007), which has severe consequences for multiple aspects of growth and development such as cognitive, linguistic, and athletic abilities. In the past, treatment of PEE has focused on ASMs and epilepsy surgery, but there has been a lack of studies on VNS for DRE after encephalitis. Since Penry first used VNS to treat DRE in 1988 (Hajnsek et al., 2005; González et al., 2019), it has been recommended for various types of epilepsy (González et al., 2019). This was the first study of PEE characterized by DRE with a follow-up period of 12 months. Twenty patients with refractory PEE underwent VNS implantation, all of whom were aged < 18 years. In this case series, VNS therapy had a clinical effect regarding seizure reduction in 17 of the 20 patients; 15 were responders (seizure reduction rate ≥ 50%), and three of them had achieved seizure freedom at 12 months. These findings were consistent with a retrospective study of five adults with refractory PEE treated with VNS (Fujimoto et al., 2018). The seizure reduction rate, effective rate, and seizure freedom rate of patients increased with time, which may be due to the time-cumulative effect of VNS (Serdaroglu et al., 2016).

Moreover, we used E-chess scores to further evaluate epilepsy severity after VNS. Statistical analysis showed that the E-chess score decreased gradually over time, and the differences between each follow-up time and baseline (before operation) were statistically significant. The results indicate that VNS can reduce the severity of epilepsy after encephalitis, and the effect is more obvious with the increase of time. We also compared patient EEG scores before and 12 months after the VNS operation. The results showed that the GTE score at 12 months was lower than that before operation, and there was a statistical difference between them, indicating that VNS can also improve the EEG of children with PEE. The postoperative GTE score of patient 7 was higher than baseline, which may be due to the decrease of two kinds of ASMs, which may also be the reason for the increase of seizure frequency.

Vagus nerve stimulation can also improve the overall quality of life and communication ability, memory, and concentration of patients with epilepsy (Wasade et al., 2015; Serdaroglu et al., 2016; Fujimoto et al., 2018). In contrast to ASMs, regardless of whether the frequency of seizures is reduced or not, VNS has been shown to have no negative effect on the cognition and mood of children, and it can even exert improvement (Klinkenberg et al., 2013). This is consistent with our findings. Fourteen of the 20 patients in our study showed an improvement in at least one of the three abilities assessed (i.e., cognitive, linguistic, and athletic). This finding is notable despite the limitation that abilities were assessed based on the subjective feelings of patients or their guardians.

Some studies have reported that the age at implantation, the course of epilepsy, and the type of epilepsy are not the main factors that influence the efficacy of VNS efficacy (Englot et al., 2016; Chrastina et al., 2018). However, there are also reports that the response to VNS may be poorer when the course of epilepsy is longer (Saneto et al., 2006; Englot et al., 2016). We compared the distribution differences between the effective group and the ineffective group at 12 months after VNS operation, and found that there were no statistical differences. This may be due to the small sample size and short follow-up time. Further analysis of the factors affecting the efficacy of VNS needs to expand the sample size.

Appropriate adjustment of the post-operative stimulation parameters is critical for obtaining optimal results. At present, there is no clear standard for setting stimulation parameters, which need to be adjusted according to the principle of individualization. There is no clear link between increased stimulation current and decreased seizures, and good results have been obtained in many patients when the current is <1.0 mA. Patient 6 achieved good seizure control at 1.0 mA, whereas patient 13 did not show much improvement even at 2.0 mA.

Vagus nerve stimulation is a minimally invasive treatment involving minor surgical trauma and rapid postoperative recovery. Adverse reactions associated with VNS therapy are usually transient manifestations caused by electrical stimulation, such as hoarseness, dysphagia, coughing, and tingling and numbness in the throat or chest (Révész et al., 2016). Although patients usually gradually adapt, these adverse reactions can also be ameliorated by reducing the stimulation parameters. In our study, patients 4 and 6 had a transient cough; none of the others had related adverse reactions. During the follow-up period, the stimulation system functioned normally for all 20 patients.

The specific mechanism of VNS in the treatment of epilepsy remains unclear. It has been confirmed that the electrical stimulation signal induced by VNS through the vagus nerve is the basis of the anti-epileptic effect (Hulsey et al., 2017). Stimulation of the vagus nerve with electrical current can affect the central nervous system, induce brain activity to desynchronize, and break the epileptic transmission network to play the role of antiepileptic (Johnson and Wilson, 2018; González et al., 2019). VNS can also increase the production of inhibitory neurotransmitters and reduce the production of excitatory neurotransmitters, playing an anti-epileptic role by stimulating the vagus nerve (Alexander et al., 2017; Johnson and Wilson, 2018; Engineer et al., 2019; González et al., 2019). It is speculated that the mechanism of VNS in the treatment of pee is related to the above mechanism. The efficacy of VNS for refractory epilepsy, in terms of reducing the frequency of epileptic seizures and improving cognitive status, has been widely recognized (Wheless et al., 2018). If the use of ASMs cannot control the seizures of patients with PEE, VNS seems to be a reasonable treatment option. However, there are few reports on VNS treatment for PEE.

Few studies have examined the treatment of refractory PEE by VNS, especially in children. To more comprehensively evaluate of the postoperative efficacy of VNS, we considered seizure reduction, the effective rate, McHugh classification, modified E-Chess scores, and GTE scores. Our results support the use of VNS in patients with PEE. The limitations of this retrospective observational study are the small sample size and relatively short follow-up period and selection bias. A multicenter clinical trial with many patients is required to further clarify the therapeutic efficacy of VNS in patients with PEE. Additional research is also needed into factors that improve VNS efficacy of PEE and the optimization of VNS parameters.

## Conclusion

We conducted a retrospective observational study on patients with PEE and found that VNS can reduce seizure frequency and severity. It also improved subjective cognitive, linguistic, and athletic abilities to varying degrees. In patients with PEE, especially children, VNS offers a potentially safe and effective treatment option without obvious adverse effects.

## Data Availability Statement

The original contributions presented in the study are included in the article/Supplementary Material. Further inquiries can be directed to the corresponding author/s.

## Ethics Statement

The studies involving human participants were reviewed and approved by the Ethics Committee of the Chinese PLA General Hospital (number/ID of approval 2017.014-1). Written informed consent to participate in this study was provided by the participants’ legal guardian/next of kin.

## Author Contributions

YS, JC, and TF wrote the first draft. YS and GY contributed to the study conception and design of the study. LW, XS, JW, ZL, and JXW acquired and analyzed the data and revised the manuscript. ZC, XX, ZL, and LZ finalized the concept, revised, and confirmed the results. All authors contributed to the critical revision of the final version of the manuscript for important intellectual content.

## Conflict of Interest

The authors declare that the research was conducted in the absence of any commercial or financial relationships that could be construed as a potential conflict of interest.

## Publisher’s Note

All claims expressed in this article are solely those of the authors and do not necessarily represent those of their affiliated organizations, or those of the publisher, the editors and the reviewers. Any product that may be evaluated in this article, or claim that may be made by its manufacturer, is not guaranteed or endorsed by the publisher.
