# Supplementary material for: Vagus Nerve Stimulation Therapy for the Treatment of Seizures in Refractory Postencephalitic Epilepsy: A Retrospective Study
Source: Front Neurosci. 2021 Aug 19;15:685685. doi: 10.3389/fnins.2021.685685 (PMC8418307; doi:10.3389/fnins.2021.685685)
Supplement: Supplementary file 1 [file Table_1.DOCX]

**Table 1. Clinical characteristics and follow-up data**

| No. | Sex | Type of EN | Age at EN onset(y) | Age at epilepsy onset(y) | The time from EN to epilepsy(y) | Age at VNS insertion(y) | Duration of epilepsy before VNS(y) | Seizure type before VNS | | ASMs before VNS | Baseline | SF  (3 m) | SF  (6 m) | SF  (12 m) |
| --- | --- | --- | --- | --- | --- | --- | --- | --- | --- | --- | --- | --- | --- | --- |
|  |  |  |  |  |  |  |  | Focal | Generalised |  |  |  |  |  |
| 1^a^ | M | VE | 0.25 | 0.33 | 0.08 | 1.58 | 1.25 |  | ES | 4 | 30-50/d | 20/d | 15/d | 15/d |
| 2^a^ | M | VE | 1.75 | 2.67 | 0.92 | 4 | 1.33 |  | ES | 3 | 20-240/d | 20-240/d | 10-80/d | 10-80/d |
| 3^c^ | M | BME | 0.01 | 2.25 | 2.24 | 5.08 | 2.83 | C | ES | 4 | 5-6/d | 2-4/d | 3-5/d | 2-4/d |
| 4* | M | BME | 1 | 1 | 0 | 9.92 | 8.92 |  | T-C | 3 | 10-90/m | 10-90/m | 10-90/m | 10-90/m |
| 5^a^ | F | VE | 0.42 | 1 | 0.58 | 4.08 | 3.08 |  | ES | 3 | 60-70/d | 40-50/d | 15-20/d | 0 |
| 6^b^* | F | VE | 8.92 | 8.92 | 0 | 11.08 | 2.16 | F to bi T-C |  | 5 | 210-240/m | 10/m | 10/m | 10/m |
| 7^c^ | M | VE | 1.08 | 1.08 | 0 | 2.17 | 1.09 | T; Em |  | 5 | 4-6/m | 0.5-1/m | 0.5-1/m | 6-10/m |
| 8 | M | VE | 2.25 | 2.33 | 0.08 | 5 | 2.67 | F to bi T-C |  | 4 | 4-8/d | 3-7/d | 4-6/d | 1-5/d |
| 9 | M | VE | 0.58 | 0.58 | 0 | 1.42 | 0.84 |  | ES; T-C | 4 | 40-50/d | 30/d | 20/d | 10/d |
| 10^d^ | F | UE | 0.71 | 0.75 | 0.04 | 2.92 | 2.17 |  | ES | 4 | 20/d | 20/d | 20/d | 10/d |
| 11^a^ | M | VE | 11 | 11 | 0 | 12 | 1 | C |  | 3 | 4-8/m | 4-8/m | 4-8/m | 4-8/m |
| 12 | M | VE | 2.08 | 3.33 | 1.25 | 4.67 | 1.34 |  | ES | 5 | 6-7/d | 5-6/d | 3-4/d | 3-4/d |
| 13^c^ | M | UE | 6 | 6 | 0 | 7 | 1 | T |  | 4 | 3-6/d | 3-6/d | 3-6/d | 3-6/d |
| 14^a^ | M | VE | 5 | 5 | 0 | 6 | 1 | C |  | 5 | 10-15/d | 10-15/d | 7-8/d | 1/d |
| 15^a^ | F | UE | 8.5 | 8.5 | 0 | 8.5 | 0 |  | T-C | 4 | 200-240/d | 0 | 0 | 0 |
| 16 | M | VE | 1.5 | 1.5 | 0 | 5 | 3.5 | T |  | 5 | 8-9/m | 4-5/m | 4-5/m | 2-3/m |
| 17^a^ | F | UE | 1.75 | 6.58 | 4.83 | 6.67 | 0.09 | Au | T-C | 5 | 100-150/d | 6-7/d | 3-5/d | 3-5/d |
| 18^cd^ | F | VE | 0.67 | 3 | 2.33 | 13 | 10 |  | T-C | 4 | 10-15/d | 20/m | 5/m | 0 |
| 19^cd^ | F | VE | 6 | 6.5 | 0.5 | 8 | 1.5 | T |  | 4 | 10-15/d | 10-15/d | 7-8/d | 3-4/d |
| 20 | M | UE | 7.5 | 12 | 3.5 | 15 | 3 |  | Ab | 4 | 20-24/m | 20-24/m | 15-20/m | 2/m |

M: male; F: female; y: year; m: month; d: day; EN:viral encephalitis; VE: viral encephalitis; BME: bacterial meningoencephalitis; UE: unknown etiology encephalitis

ES: epileptic spasms; C: clonic; T-C: tonic-clonic; F to bi T-C: focal to bilateral tonic-clonic; T: tonic; Em: emotional; Au: autonomic; Ab: absence; SF: seizure frequency

^a^Improved cognitive, linguistic, and athletic abilities; ^b^Improved cognitive and linguistic abilities; ^c^Improved cognitive ability only; ^d^Improved athletic ability only; *With cough
