# Supplementary material for: Vagus Nerve Stimulation Therapy for the Treatment of Seizures in Refractory Postencephalitic Epilepsy: A Retrospective Study
Source: Front Neurosci. 2021 Aug 19;15:685685. doi: 10.3389/fnins.2021.685685 (PMC8418307; doi:10.3389/fnins.2021.685685)
Supplement: Supplementary file 2 [file Table_2.DOCX]

**Table 2. Distribution difference between effective group and ineffective group at 12 months after VNS**

|  | Classification | Effective group | Ineffective group | P value |
| --- | --- | --- | --- | --- |
| Sex | M | 7 | 6 | 0.051 |
|  | F | 7 | 0 |  |
| Type of encephalitis | VE | 10 | 3 | 0.093 |
|  | BME | 0 | 2 |  |
|  | UE | 4 | 1 |  |
| Seizure type before VNS | Generalised | 8 | 2 | 0.502 |
|  | Focal | 5 | 3 |  |
|  | Generalised and focal | 1 | 1 |  |
| Age at encephalitis onset (y) |  | 1.75（0.65，6.38） | 1.58（0.75，7.25） | 0.967 |
| Age at epilepsy onset (y) |  | 4.26±3.70 | 4.11±3.85 | 0.935 |
| Age at VNS insertion (y) |  | 6.59±4.13 | 6.81±3.63 | 0.912 |
| The time from encephalitis to epilepsy (y) |  | 0.08（0，1.27） | 0（0，1.50） | 0.387 |
| Duration of epilepsy before VNS (y) |  | 1.83（0.96，3.02） | 1.22（1.00，4.35） | 0.869 |
| VNS intensity (mA) |  | 1.79±0.40 | 1.70±0.37 | 0.661 |

M: male; F: female; VE: viral encephalitis; BME: bacterial meningoencephalitis; UE: unknown etiology encephalitis
